# Development of immunogold test strips for chilli veinal mottle virus detection using monoclonal antibodies against the CP

**DOI:** 10.3389/fmicb.2025.1721102

**Published:** 2025-12-17

**Authors:** Fang Wang, Chuantao Xu, Ying Chen, Ke Wang, Xueying An, Benguo Zhou

**Affiliations:** 1 Anhui Academy of Agricultural Sciences, Hefei, China; 2 Anhui Province Key Laboratory of Pesticide Resistance Management on Grain and Vegetable Pests, Hefei, China; 3 Institute of Luzhou Tobacco Company of Sichuan Province, Luzhou, China; 4 Xuancheng City Xuanzhou District Tobacco Industry Development Center, Xuancheng, China

**Keywords:** monoclonal antibodies, immunochromatographic strip, tobacco plants, sandwich, chilli veinal mottle virus

## Abstract

Chilli veinal mottle virus (ChiVMV), a member of the *Potyviridae* family, a major threat to solanaceous crops, including tobacco and peppers, in China, where it causes systemic necrosis, leaf mottling, and significant yield losses. Early and accurate detection is essential for effective disease management, yet conventional assays such as RT-PCR and ELISA are constrained by laboratory requirements and skilled personnel. In this study, we developed a colloidal gold-based immunochromatographic strip (ICS) for the rapid, specific, and field-deployable detection of ChiVMV. The viral coat protein (CP) was heterologously expressed in *Escherichia coli* and used to generate eight hybridoma cell lines, from which two monoclonal antibodies, 22F4 (capture) and 45C4 (detector), were selected. The assembled ICS yielded clear visual results within 5–10 min, with a detection limit corresponding to a 1:1,000 dilution of infected leaf extracts, and exhibited no cross-reactivity with PVY, TVBMV, WYMV, TMV, or CMV. Field testing with 120 tobacco samples demonstrated 95.7% sensitivity and 98.0% specificity, consistent with RT-PCR, and the test strips maintained stability after 12 months of storage at room temperature. This study presents the first rapid ICS for ChiVMV, providing a sensitive, specific, and cost-effective diagnostic tool for on-site disease surveillance and early management in tobacco cultivation.

## 1 Introduction

Chilli veinal mottle virus (ChiVMV), a member of the *Potyviridae* family and Potyvirus genus, has recently emerged as a virus affecting tobacco in China (Jiao et al., 2020). In recent years, this virus has been reported in various tobacco-growing regions, exhibiting potential for significant damage and showing signs of becoming a major disease (Yang et al., 2013). The virus spreads rapidly and poses a significant threat to the healthy cultivation of solanaceous crops, including peppers and tobacco (Shah et al., 2008).

ChiVMV is highly destructive to its host, with symptoms exhibiting considerable variation across different plant species (Lakshminarayana Reddy et al., 2024). In tobacco, the virus induces systemic leaf necrosis, resulting in mottling, yellowing, and curling of the leaves. As the virus accumulates, it causes the tobacco leaves to dry out and fall off, beginning with the lower leaves, and this may ultimately lead to the death of the entire plant (Shouhui et al., 2024). ChiVMV was first reported in 1979, affecting peppers in Malaysia, and it has since spread, causing significant damage in various countries and regions worldwide (Rao et al., 2020). In China, the virus has rapidly spread in pepper- and tobacco-growing regions across multiple provinces, including Shaanxi, Hainan, Yunnan, Guangxi, Sichuan, Chongqing, Guangdong, Guizhou, Hunan, and Fujian (Ding et al., 2011; Shouhui et al., 2024; Yang et al., 2013). It has become a dominant virus affecting solanaceous crops in China, severely limiting their production. Therefore, to control the occurrence and spread of ChiVMV and ensure accurate and scientific management, effective virus diagnosis is essential. In addition, the disease cannot be accurately detected during the early stage of plant growth and is thus easily misdiagnosed, leading to serious outbreaks during the harvest season. Therefore, a fast, accurate, and simple detection method for ChiVMV is urgently needed. Currently, many types of assays have been used to detect ChiVMV, such as reverse transcription polymerase chain reaction (RT-PCR) (Sanabam et al., 2018), enzyme-linked immunosorbent assay (ELISA) (Jiao et al., 2020), reverse transcription loop-mediated isothermal amplification (RT-LAMP) (Banerjee et al., 2016), real-time RT-PCR, and optical coherence tomography (Gong et al., 2023). Although these methods can achieve accurate detection results, they are not suitable for the online screening of samples on a large scale because of the complex requirements regarding sample pretreatment and operating techniques, as well as the lengthy analysis times (Zhang et al., 2021). Thus, there is a growing need for viable, economical, and rapid methods that can detect ChiVMV accurately.

The immunochromatographic assay (ICA), which relies on the specific binding between antigens and antibodies, has been increasingly used for both qualitative and quantitative analysis, as well as for rapid screening of various samples (Li et al., 2025). Compared with traditional methods such as ELISA, ICA is faster, easier to perform, more cost-effective, and generally exhibits good stability (Carra et al., 2009; Murugananthan et al., 2018). These advantages have led to the widespread use of immunochromatographic strips (ICSs) in on-site and point-of-care testing across a range of fields, including the detection of pesticides, heavy metals, toxins, proteins, hormones, pathogens, and drugs (Zhang et al., 2024). For example, ICA has been used to detect tylosin and tilmicosin in milk, with researchers finding a cut-off value of 8 ng/mL for tylosin (Wang et al., 2021). Similarly, ICS has been applied to identify imidacloprid in agricultural and environmental samples, achieving a detection limit of 0.45 ng/mL (Lu et al., 2021). In clinical settings, ICS is also utilized to screen for cryptococcal antigens in serum and cerebrospinal fluid samples (Wang et al., 2020).

There has been significant progress regarding the application and development of the immunochromatographic assay for plant virus detection in recent years. For example, Bin (Bin et al., 2018) developed an ICS to detect the citrus yellow vein clearing virus (CYVCV), which was able to detect CYVCV from tissue extracts at a ratio of 1:320 (w/v). ICS tests have been used to diagnose many plant diseases, including those caused by citrus tristeza virus (CTV) (Salomone et al., 2004), tobacco mosaic virus (TMV) (Drygin et al., 2009), and soybean mosaic virus (SMV) (Zhu et al., 2016). However, to date, there are limited reports on the development of colloidal gold ICS assays specifically targeting ChiVMV, highlighting the need for further research in this area.

The CP of ChiVMV is the primary structural protein. It is highly immunogenic and relatively conserved, enabling it to stimulate the production of antibodies during the early stages of infection (Tsai et al., 2008). Based on the CP, a range of serological and molecular detection methods have been developed to identify viral infections (Shukla and Ward, 1989). However, research on the development of monoclonal antibodies against tobacco ChiVMV remains limited. In this study, we expressed and purified the CP of ChiVMV. Through mouse immunization and hybridoma technology, we screened two monoclonal antibodies with strong specificity against the CP. Additionally, we developed a colloidal gold immunochromatographic test strip, which provides a convenient diagnostic tool for ChiVMV detection.

## 2 Materials and methods

### 2.1 Expression and purification of ChiVMV coat protein (CP)

The preparation method was adapted from previously published protocols (Banerjee et al., 2016; Su et al., 2021) with slight modifications. Total RNA was isolated from ChiVMV-infected tobacco leaves using Trizol reagent (Takara, Japan). After removing genomic DNA contamination, RNA samples were converted into complementary DNA (cDNA) using 5× Hiscript qRT Supermix (Vazyme, China). Specific primers were designed based on the published ChiVMV polyprotein sequence (accession number AGN92430.1). The forward primer (F: TAATACGACTCACTATAGGGGCAGGAGAGAGTGTTGA) and reverse primer (R: TGCTAGTTATTGCTCAGCGGCATAATCCCCGAACGCC) included the restriction sites NdeI and EcoRI, respectively. Additionally, a GS linker and a 6× His-tag were attached at the N-terminal region. The amplified fragment was then digested and inserted into a pET28b vector via the corresponding NdeI and EcoRI restriction sites, creating the recombinant construct pET28b-CP. Subsequently, purified recombinant plasmid was introduced into *Escherichia coli* BL21 competent cells for expression of the recombinant protein. The resulting purified protein served as an antigen for generating antibodies against ChiVMV (Park et al., 2021; Volkova et al., 2021).

### 2.2 Preparation of the monoclonal antibody against ChiVMV

The purified immunogen was diluted in PBS (0.15 M, pH 7.0) to a final concentration of 2 mg/mL. For initial immunization, the immunogen was thoroughly emulsified with an equal volume of Freund’s complete adjuvant. Five female BALB/c mice (aged 6 weeks) were immunized, each receiving a subcutaneous injection of 200 μL of the mixture. Subsequent immunizations utilized Freund’s incomplete adjuvant instead. The second immunization was administered 3 weeks after the first, followed by additional injections every 2 weeks, totaling five immunizations. Beginning with the third injection, antiserum samples were collected from the tail vein 7–10 days post-immunization to determine antibody titers using indirect ELISA as described by Miura et al. (2008). The mouse showing the highest antibody titer was selected as the donor for spleen cells.

Seven days before hybridoma preparation, SP2/0 myeloma cells were thawed and cultured in DMEM supplemented with 20% fetal bovine serum. The murine myeloma cell line SP2/0, used for hybridoma generation, was maintained in our laboratory under standard culture conditions. Three days prior to fusion, the selected donor mouse received an intraperitoneal booster injection containing 200 μL of immunogen without adjuvant. Spleen cells were then harvested by isolating the spleen, mechanically disrupting it, and centrifuging the resulting cell suspension. Subsequently, spleen cells were uniformly mixed with SP2/0 myeloma cells, and PEG1500 was added gradually over 1 min to facilitate fusion. The reaction was promptly stopped using DMEM. The fused cells were resuspended in complete culture medium and plated uniformly into 96-well plates, before being cultured at 37 °C under 5% CO_2_. After 7 days, indirect ELISA screening, utilizing 3,3′,5,5′-tetramethylbenzidine (TMB) substrate, identified wells containing hybridomas secreting antibodies of interest. Positive cells were then further selected via HAT (DMEM containing 10% FBS, 100 μM hypoxanthine, 0.4 μM aminopterin, and 16 μM thymidine) and HT (DMEM containing 10% FBS, 100 μM hypoxanthine, and 16 μM thymidine) media (Sigma-Aldrich, China), followed by multiple rounds of subcloning through limiting dilution to ensure monoclonal stability.

Stable hybridoma clones producing high-titer antibodies were ultimately selected; cryopreserved using a solution composed of complete growth medium, fetal bovine serum, and DMSO; and stored in liquid nitrogen. Ascitic fluid collected from mice was purified by affinity chromatography, yielding monoclonal antibodies of high purity, stored at −80 °C. Additionally, unpurified ascites was mixed with 50% glycerol and preserved at −20 °C for subsequent use.

### 2.3 Western blotting

Eukaryotic expressed CP samples were used for the positive samples, and buffers were used as negative samples. Protein samples were collected and subjected to Western blot analysis, 100 ng of the purified recombinant ChiVMV CP was mixed with 4× SDS loading buffer. The protein sample was separated by electrophoresis in 12% SDS-polyacrylamide (SDS-PAGE) gel after boiling for 10 min and transferred onto an Nitrocellular (NC) membrane (Whatman, Dassel, Germany) conducted using CP-specific monoclonal antibody ascitic fluid as the primary antibody and HRP-conjugated goat anti-mouse IgG as the secondary antibody. After ECL chemiluminescent detection, the results were observed and recorded using a UVP imaging system.

### 2.4 Preparation of the Nanogold–mAb probe

The preparation of the nanogold probe combined with the mAb was based on the process described by Byzova et al. (2018), with minor modifications. Initially, the pH of the colloidal gold solution was adjusted to 8.2 with 0.1 M K_2_CO_3_, and 3.2 mg/mL of the solution was added to 10 mL of the pH-adjusted colloidal gold solution. The solution was then mixed vigorously for 5 min and left standing at room temperature for 2 h. We ensured that the addition of the mAb was 10% more than the optimal reference amount. Filter-sterilized 10% BSA was then added to a final concentration of 1% and rapidly mixed for 5 min. After standing at room temperature for 1.5 h, the mixture was centrifuged (10,000 rpm) at 4 °C for 30 min, and the supernatant was removed carefully. Finally, the obtained sediment was suspended with 0.02 M Tris–HCl buffer containing BSA (1.0%), sucrose (1.0%), and Tween-20 (0.25%) and stored at 4 °C for future study in 20 days.

### 2.5 Indirect ELISA

Indirect ELISA was performed as described by Morozova et al. (2005) with minor modifications (Morozova et al., 2005). Flat bottomed polystyrene microtitre plates (Corning, New York, USA) were coated with crude extracts from ChiVMV-infected leaves and incubated at 4 °C overnight. The coated plates were washed once with PBST (PBS containing 0.05% (v/v) Tween-20, pH 7.4) and blocked with 200 μL of 1% BSA in PBS (at 37 °C) for 1 h. After one wash, 50 μL of antiserum diluted with 1% BSA in PBST (1:100–1:320,000) was added to each well of the plates; then, the plates were incubated for 3 h at 37 °C. After a washing step, 50 μL of alkaline phosphatase-conjugated secondary antibody (Sigma, Shanghai, China) (1:100,000 in 1% BSA in PBST) was added to each well of the plates, and the plates were then incubated at 37 °C for 3 h. The plates were washed three times, and then 50 μL of the 1 mg/mL pNPP-Na (Sangon, Shanghai, China in substrate buffer (10% diethanolamine and 0.004% MgCl_2_)) was added to each well of the plates for color development. The color development was stopped with 0.1 M EDTA (50 μL per well, pH 7.5). After 10–20 min, the absorbance was measured in single-wavelength mode at 405 nm.

### 2.6 Assembly of the ICS test

The ICS test consists of five parts: a sample pad, a bonding pad, a nitrocellulose membrane, an absorbent pad, and a PVC plate. The nanogold–mAb probe was evenly distributed onto the bonding pad (containing 7.5% EDTA) using XYZ-3060 and dried. The sample pad was treated with 0.01 M PBS containing 0.2% Tween-20. The capture antibody (containing 3% sucrose) was diluted with 0.01 M PBS and evenly sprayed onto the T line, while 0.5 mg/mL of the goat anti-mouse IgG dilution was evenly dispensed onto the C line. The nitrocellulose membrane was placed in a vacuum oven at 37 °C and vacuum-dried for 50 min. The distance between the T and the C lines was approximately 5 mm. The sample, bonding and absorbent pads, and nitrocellulose membrane were prepared, attached to the PVC plate in the correct order, and cut into 3 mm wide test strips using a CM4000 slitter. Prepared test strips were sealed in a plastic bag with a desiccant, vacuumed-dried, and stored at room temperature.

### 2.7 The immunochromatographic assay

The ICS assay process is as follows: A 100 μL liquid sample is placed onto the sample pad. The sample then migrates by capillary action through the test strip. Samples containing ChiVMV particles react with nanogold-combined 22F4 in the colloidal gold bonding pad. The nanogold–22F4–ChiVMV complex then migrates into the nitrocellulose membrane and reacts with mAb 45C4 on the T line to form the nanogold-combined 22F4–ChiVMV–45C4 sandwich complex, which is visibly observed as a red band. The nanogold–22F4 combination without ChiVMV particles migrates past the T line and reacts with goat anti-mouse IgG on the C line to form the nanogold–22F4–IgG complex, producing the second visible red band. The remaining part of the solution moves along the strip and finally accumulates in the absorption pad. In tested samples that do not contain ChiVMV, the T line does not develop a color, but the C line typically develops a color.

### 2.8 Evaluation of the ChiVMV ICS

#### 2.8.1 Specificity evaluation

The specificity of the ChiVMV colloidal gold test strip was assessed by testing against other common plant viruses, such as potato virus Y (PVY), tobacco vein banding mosaic virus (TVBMV), wheat yellow mosaic virus (WYMV), cucumber mosaic virus (CMV), and TMV, to ensure no cross-reactivity.

#### 2.8.2 Sensitivity evaluation

The sensitivity of the ChiVMV ICS was evaluated by serially diluting the positive control sample with PBS to final dilution ratios of 1:50, 1:100, 1:500, 1:1,000, 1:2,000, and 1:5,000. For each dilution, 100 μL of the sample solution was applied to the bonding pad of the test strip. A negative control sample was used as a reference. The ICSs were incubated at room temperature for 10 min, and the results were observed and recorded.

#### 2.8.3 Sensibility evaluation

Tobacco samples infected with Chili Veinal Mottle Virus (ChiVMV) were collected and subjected to RT-PCR. The RT-PCR method followed the protocol described in a previous study (Varkonyi-Gasic et al., 2007). The primers used were ChiVMV-CP-F (GCAGGAGAGAGTGTTGATGC) and ChiVMV-CP-R (CAATCCTCGAACGCCCAGCA). The reaction system consisted of the following components: 12.5 μL of 2× PCR Mix, 1 μL each of 10 μmol/L forward and reverse primers, 2 μL of cDNA, and double-distilled water added to a total volume of 25 μL. The PCR amplification program was as follows: 94 °C for 3 min, 34 cycles of denaturation at 94 °C for 30 s, annealing at 58 °C for 30 s, extension at 72 °C for 60 s, and extension at 72 °C for 10 min. The PCR products were analyzed using 1% agarose gel electrophoresis. The same samples were detected using the ChiVMV ICS to evaluate the sensibility of the ICS.

#### 2.8.4 Stability evaluation

The prepared test strips were stored under conditions at room temperature (22–25 °C). Samples were taken at 1, 3, 6, 9, and 12 months after storage to evaluate stability. Both positive control samples and negative control samples were tested at each time point, and the results were observed to determine whether any changes occurred in the detection performance of the ICS over time.

## 3 Result

### 3.1 Expression and purification of ChiVMV CP in *E. coli*

**Figure 1:**
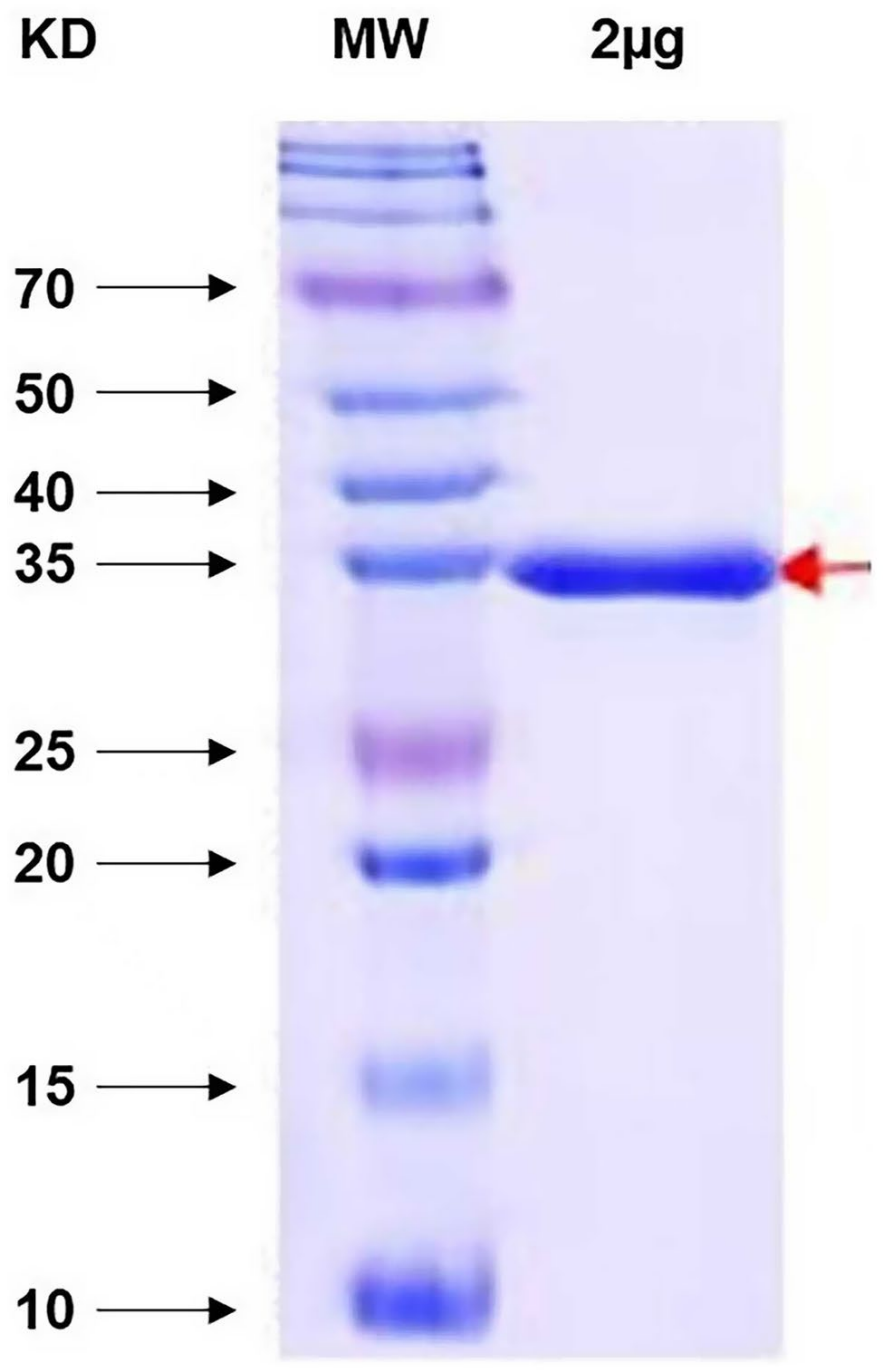
Photographs of an SDS-PAGE gel following separation of recombinant proteins, the position of a 35 kDa recombinant CP protein is indicated by an arrow. M: Protein size marker.

To obtain large amounts of ChiVMV antigens for immunization, we expressed and purified the ChiVMV CP in *E. coli*. The full-length ChiVMV CP was cloned into pET28a, and the positive clone was introduced into *E. coli* strain BL21 construction of the recombinant plasmid pET28b-CP. After IPTG induction, the N-terminal 6xHis-tagged recombinant ChiVMV CP protein was purified by Ni-NTA resin and separated on a 10% SDS-PAGE gel. Expression of a predicted 35 kDa protein was observed in the fraction eluted using 250 mM imidazole buffer (Figure 1). In total, we obtained 1 mg (1 mg/mL in a 1 mL volume) of the ChiVMV CP protein, suggesting that we can obtain an adequate amount of recombinant ChiVMV CP protein for immunization using the prokaryotic expression system.

### 3.2 Preparation of monoclonal antibodies for ChiVMV CP

**Figure 2:**
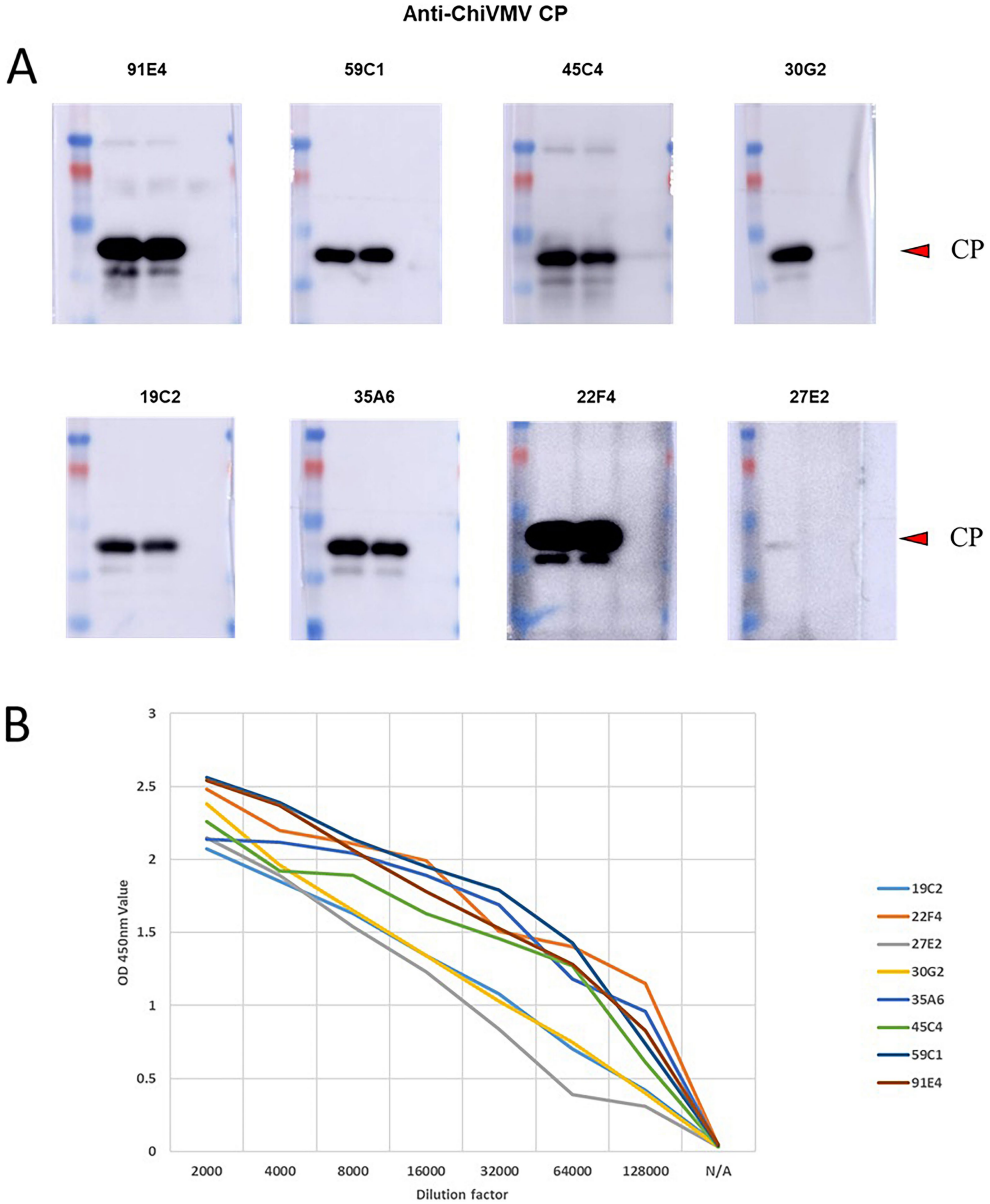
**(A)** Western blot analysis detected eight monoclonal antibodies that specifically recognized the CP protein. **(B)** Titer determination of mice producing ChiVMV monoclonal antibody.

To generate monoclonal antibodies for ChiVMV CP, five 6-week-old BALB/c female mice were immunized with purified ChiVMV CP. One week after the third immunization, blood was collected from the mice, and the antibody titer was determined to be 1:128,000, which met the fusion requirements. Spleen cells were then fused with SP2/0 cells, and indirect ELISA was used for positive screening. After subcloning, eight hybridoma cell lines were obtained: 19C2, 22F4, 27E2, 30G2, 35A6, 45C4, 59C1, and 91E4. Western blot analysis showed that all eight monoclonal antibodies specifically recognized the CP (Figure 2A). Indirect ELISA testing revealed that the ascitic fluid antibody titers for all eight clones were greater than 1:128,000 (Figure 2B). After cell fusion and subcloning, eight hybridoma cell lines that stably secrete the ChiVMV monoclonal antibody were screened. After antibody subtype identification using an ISO StripTM Monoclonal Antibody Isotyping Kit (Roche, Switzerland), mAbs 19C2 was IgG1; mAbs 2C9, 22F4, 27E2, 45C4, 91E4, and 30G2 were IgG2a; and mAbs 35A6 and mAbs 59C1 were IgG2b. All light chains were kappa chains. Western blot analysis (Figure 2A) and indirect ELISA (Figure 2B) confirmed that all eight monoclonal antibodies reacted with ChiVMV CP, and 22F4 and 45C4 produced the strongest and most specific signals with no nonspecific bands. In indirect ELISA, these two antibodies also exhibited the highest OD_450_ nm values at high dilution factors, demonstrating superior affinity. Additionally, IgG2a and IgG2b subclasses possess higher Fc stability and stronger antigen-binding activity than IgG1, which makes them particularly suitable for capture–detection pairing in immunochromatographic strips. Therefore, 22F4 (IgG2a) was used as the capture antibody and 45C4 (IgG2a) as the detection antibody. We then chose the monoclonal antibodies 22F4 and 45C4 for further experiments.

### 3.3 Preparation of colloidal gold-labeled monoclonal antibody

**Figure 3:**
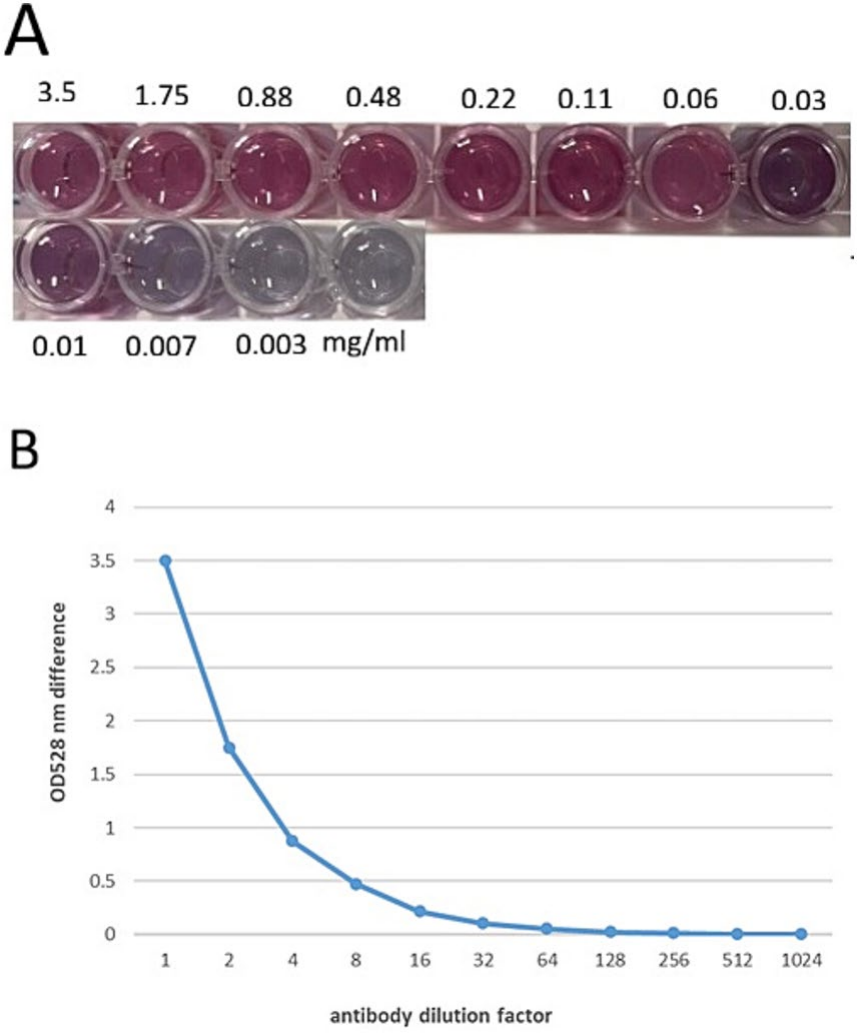
**(A)** Optimizing the ratio of colloidal gold and monoclonal antibody for conjugation. A fixed amount of colloidal gold (125 μL) was mixed with an increasing amount, from 0 to 3.5 mg/mL (30 μL) of mAb 22F4. As the concentration of the antibodies increased, the color of the conjugated antibody-colloidal gold became pink. **(B)** Flocculation curve of monoclonal antibody 22F4.

Colloidal gold was then prepared by a classical method using trisodium citrate to reduce the chloroauric acid (at a ratio of 1:1.2 chloroauric acid and sodium citrate) (Wang et al., 2014). NaCl, as a strong electrolyte, can disrupt the surface charge of colloidal gold, leading to its aggregation. This property can be utilized to evaluate whether colloidal gold has fully reacted with antibodies by adding NaCl solution into the wells. As shown in Figure 3A, upon addition of 10% NaCl solution to the colloidal gold solution in the wells, a color change was observed in some wells compared to their appearance prior to NaCl addition. The antibody dilution factor increases from left to right across the wells. We also used UV absorption (OD528nm) as an alternative method to determine the ratio of antibodies and colloidal gold for conjugation. When the concentration of the 22F4 antibody decreased from 0.219 to 0.027 mg/mL, the absorption curve dropped, and when it decreased further from 0.219 to 0.027 mg/mL, the absorption curve was nearly smooth. (Figure 3B). The well showing minimal color change and the smallest difference in absorbance before and after NaCl addition is considered the critical point at which the colloidal gold-antibody complex remains stable. The antibody dilution at this point indicates the optimal amount of antibody required for stabilizing the colloidal gold during labeling. As observed in Figure 3A, with increasing antibody dilution, the color of the wells after NaCl addition initially deepens, then fades, and ultimately disappears. The deepest color, indicating maximal binding between antibody and colloidal gold, was observed at a 64-fold dilution. At a 128-fold dilution, the solution retained a cherry-red color, whereas further dilutions resulted in noticeable discoloration. In conjunction with the antibody aggregation curve, it was found that at a 128-fold dilution, the OD528 difference was small and the curve approached a plateau, indicating that this is the maximum dilution at which the antibody can still maintain the stability of the colloidal gold system. Given that the initial antibody concentration was 3.5 mg/mL, the concentration at a 128-fold dilution is 0.027 mg/mL. Therefore, adding 27 μg of antibody to 1 mL of colloidal gold solution is sufficient to stabilize the system.

### 3.4 Assembly of ICS and rapid detection of ChiVMV

**Figure 4:**
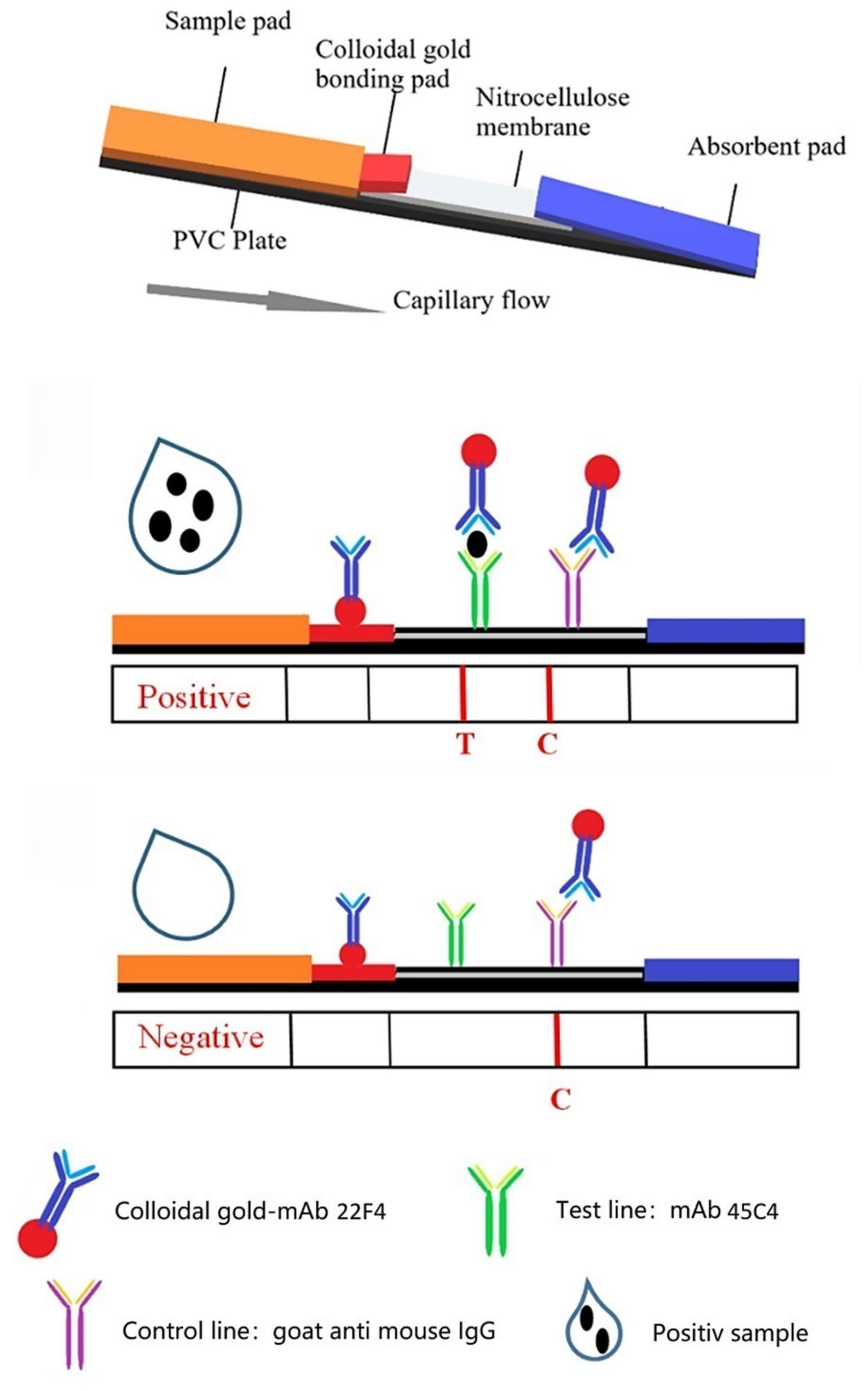
Characterization of the test result.

**Figure 5:**
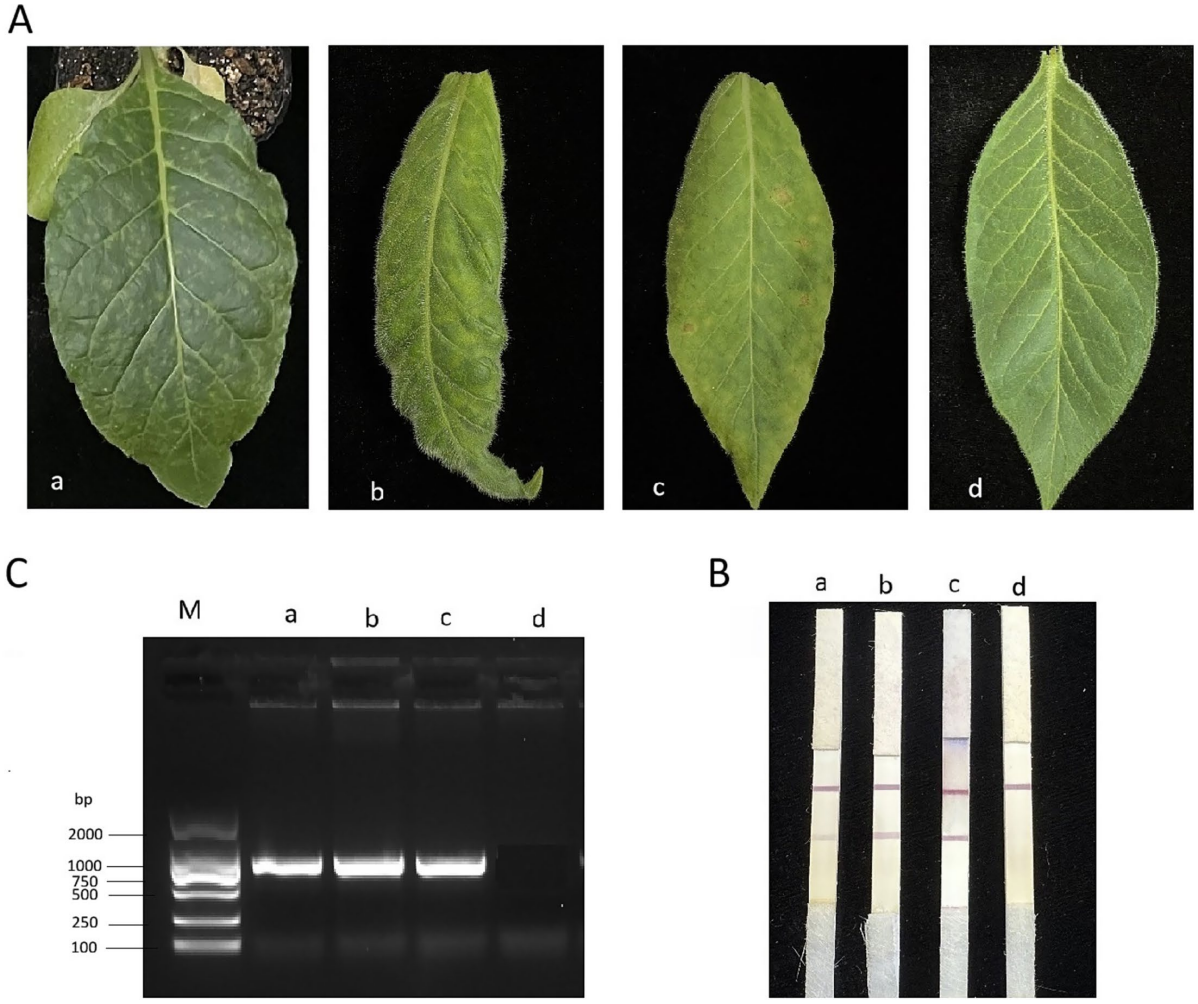
**(A) (a–c)** Inoculated ChiVMV tobacco leaves. **(D)** Healthy tobacco leaves. **(B)** Detection using the ICS. **(C)** PCR detection of samples a, b, c, d.

We assembled the ICS by sequentially adding a sample absorbing pad, an application pad with conjugated mAb 22F4-colloidal gold, a Millipore 180 nitrocellulose membrane and a water absorbing pad. To capture the ChiVMV-conjugated mAb 22F4-colloidal gold complexes, another clone of mAb 45C4 was purified and coated at the test line on the nitrocellulose membrane. The test line will turn pink if the tobacco leaf sample contains ChiVMV. Protein A was coated at the control line on the nitrocellulose membrane, which captured all conjugated antibody-colloidal gold to demonstrate that antibody coated gold flowed along the ICS (Figure 4). To test the assembled ICS, ChiVMV-infected or healthy leaves were ground into PBS and 150 μL of supernatant was added to the sample pad. As shown in Figures 5A,B, within 5–10 min, the test line became clearly pink in color with the addition of an ChiVMV-infected sample, whereas the color of the test line remained unchanged with the addition of a healthy sample. The control line of both samples turned pink, suggesting that the strip works well (Figure 5).

### 3.5 Specificity evaluation of the ICS test

**Figure 6:**
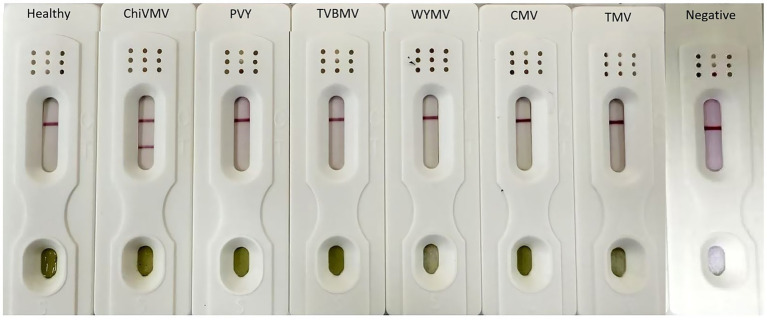
Photographs of ICS used to test samples infected with ChiVMV, TMV, CMV, WYMV, PVY, and TVBMV; a healthy plant was used as the negative control.

Specificity is a critical aspect when detecting ChiVMV. In this study, we tested tobacco samples infected by ChiVMV, alongside PVY, TVBMV, WYMV, TMV, and CMV. The results showed that the C line and T line of the tested samples from the different plants infected by ChiVMV developed a color response, whereas samples from leaves infected with PVY, TVBMV, WYMV, TMV, and CMV showed no color at the T line. As expected, the C line developed for all samples. Therefore, no cross-reaction with viruses from the same genus, including PVY, TVBMV, WYMV, CMV, and TMV, was identified. These results indicate that the ICS for detecting ChiVMV has acceptable specificity (Figure 6).

### 3.6 Sensitivity evaluation of the ICS test

**Figure 7:**
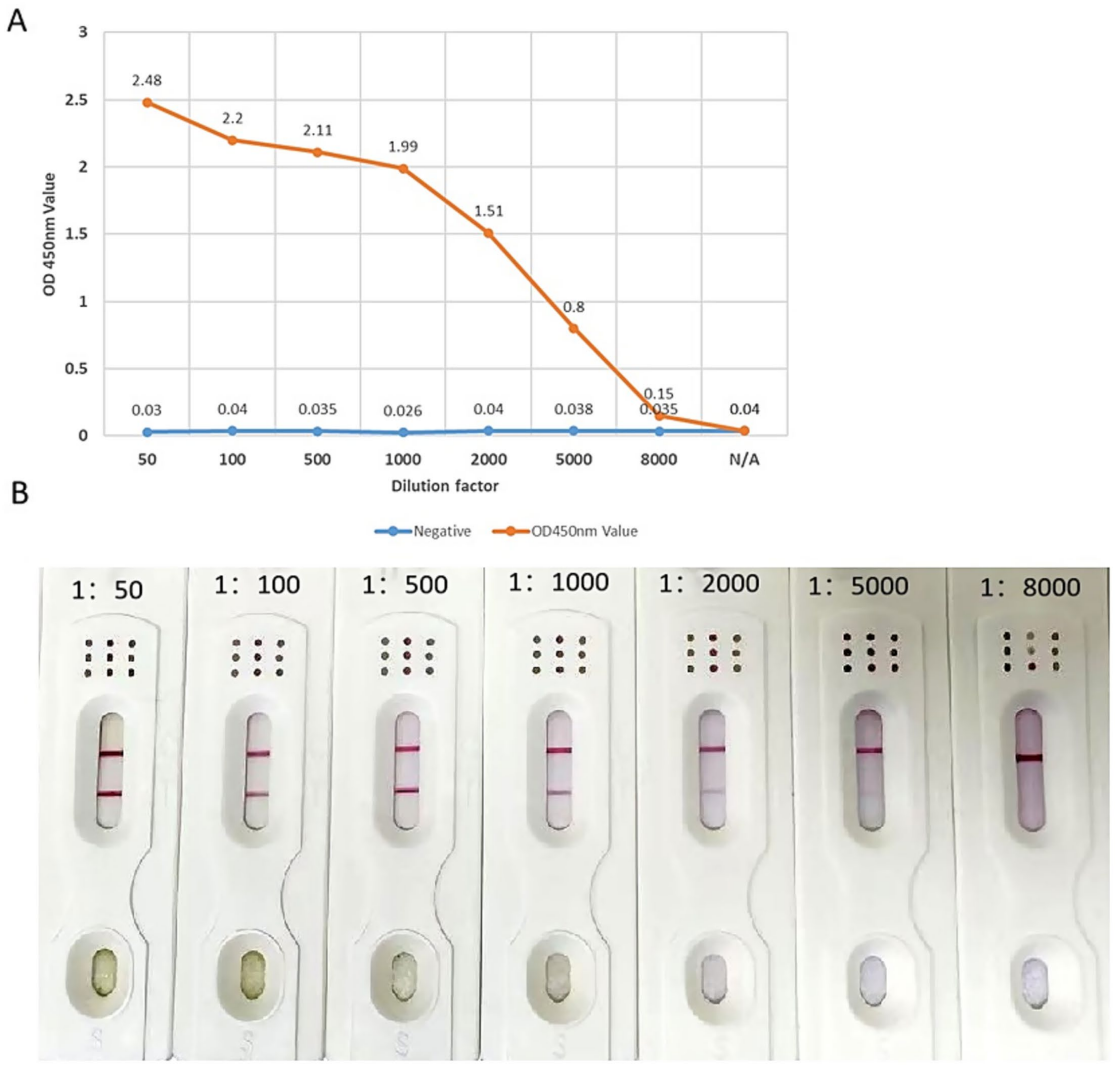
Sensitivity comparison of ICS and ELISA for the detection of ChiVMV. **(A)** The ChiVMV-infected leaf samples diluted from 1:50 to 5,000 were tested using the indirect ELISA. **(B)** Photos of ICS used to test dilutions of ChiVMV infected leaves. A healthy leaf sample was used as the negative control.

To examine the sensitivity of the ICS, the ChiVMV-infected leaves were ground in PBS at a ratio of 1 g: 1 mLPBS and then diluted from 1:50, 1:100, 1:500, 1:1,000, 1:2,000, and 1:5,000. One hundred fifty microliters of each dilution was added to the sample pad of the ICS. A healthy tobacco sample was used as a negative control. When the sample was diluted from 1:50 to 1:1,000, the test line of the ICS gave a clear positive band. At a 1:2,000 dilution, the result was ambiguous, and at a 1:5000 dilution, the result was negative. These results suggest that the ICS can detect a positive signal with a 1,000-fold dilution for the ChiVMV leaf sample (Figure 7B). To compare the sensitivity between ELISA and the ICS method, an indirect ELISA was performed using the anti-ChiVMV mAbs. The indirect ELISA gave a positive result when the ChiVMV-infected leave extract was diluted 2,000 folds (Figure 7A).

### 3.7 Sensibility evaluation of the ICS (field sample testing)

The accuracy of the ICS test was evaluated by taking tobacco field samples from Xuancheng City, Anhui Province; Hengyang City, Hunan Province; and Luzhou City, Sichuan Province. Tobacco leaves were collected (Figure 5A). Plant samples of about 1 × 1 cm were placed into a grinding bag, 1 mL working buffer (0.01 M PBS (pH 7.4) containing 0.2% Tween-20 and 1% BSA) was added,and the sample was gently ground until the grinding solution changed color. Three to four drops of the ground solution were applied to the test strip, and a change in color was monitored (Figure 5B). Samples were also taken for PCR analysis (Figure 5C). The detection results derived from using the two methods were consistent for the diseased and healthy samples. Both assays detected the diseased sample but did not detect the healthy sample.

### 3.8 Stability evaluation of the ICS test

**Figure 8:**
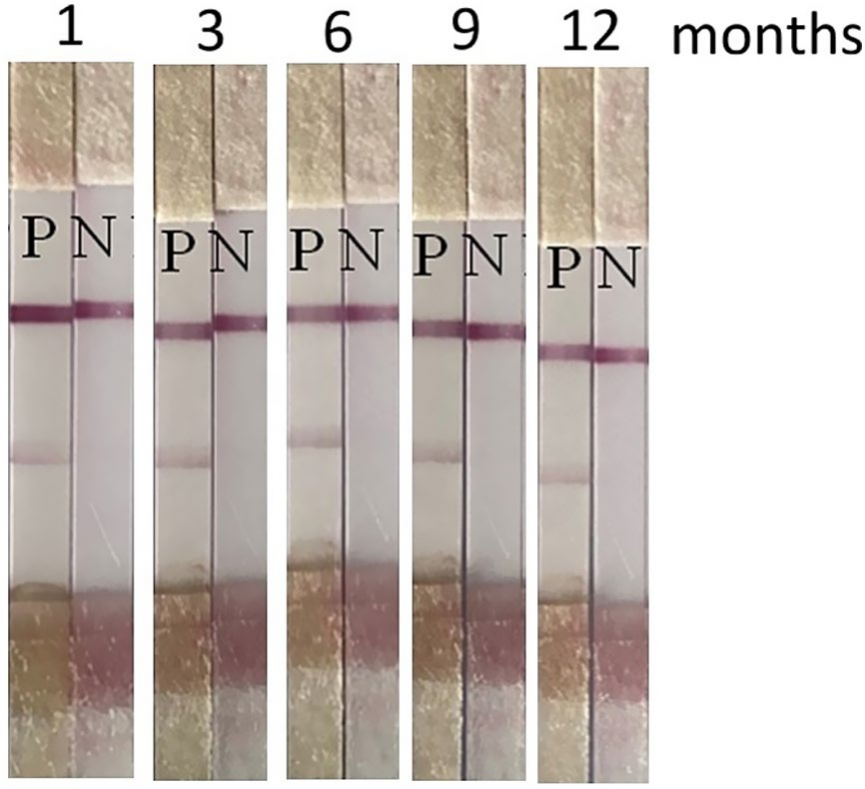
Stability of the ChiVMV immunochromatographic test strip after storage for 1, 3, 6, 9, and 12 months at room temperature (P, positive control; N, negative control).

ICS tests prepared in the same batch were placed to dry at room temperature, and the stability of the strips was tested at intervals of 1, 3, 6, 9, and 12 months. Five test groups corresponded to the five storage periods. Two bands indicate positive results, and one band indicates negative results. No loss of signal intensity was observed during the 12-month tests. The results showed that the test strip maintained accurate detection of healthy and diseased leaves after storage for an extended period. Therefore, the test strip has acceptable stability (Figure 8).

## 4 Conclusion

This study presents the first rapid-test immunochromatographic strip (ICS) that reliably identifies ChiVMV directly in the field. By cloning and heterologously expressing the viral coat protein (CP) gene in *Escherichia coli*, we obtained a high-purity recombinant antigen that was used to generate two mouse monoclonal antibodies, 22F4 and 45C4. Both antibodies bound the CP with nanomolar affinity and showed no detectable cross-reactivity to the five most common tobamoviruses and potyviruses.

Using 22F4 as the capture reagent (immobilized on the test line) and 45C4 conjugated to 40 nm colloidal gold particles as the detector, the assembled strip returned an intense, unambiguous test line within 8 min for samples containing ≥10 ng mL^−1^ of viral protein—comparable to laboratory ELISA but without specialized equipment. Healthy tissue, mock-inoculated controls, and plants infected with unrelated viruses all produced a single control line, confirming the assay’s specificity. The strips diagnostic sensitivity and specificity, calculated from 120 field samples (70 symptomatic, 50 asymptomatic), were 95.7 and 98.0%, respectively, and RT-PCR registered a value of 94 and 96%, indicating almost perfect agreement (Table 1).

**Table 1:** Comparison of ChiVMV detection results between ICS and RT-PCR methods.

Detection method	No. of symptomatic samples tested	No. of asymptomatic samples tested	Positive results	Negative results	Sensitivity (%)	Specificity (%)	Overall agreement (%)
ICS	70	50	67	49	95.7	98.0	96.7
RT-PCR	70	50	66	48	94.3	96.0	95.0

The ICS method correctly identified 67 out of 70 symptomatic (ChiVMV-infected) samples and 49 out of 50 asymptomatic (healthy) samples.

Diagnostic sensitivity = TP/(TP + FN) × 100%.

Diagnostic specificity = TN/(TN + FP) × 100%. TP, True Positive; FN, False Negative; TN, True Negative; FP, False Positive.

RT-PCR results were used as the gold standard for validation.

The strip enables growers and extension officers to confirm ChiVMV infections during routine scouting, accelerate rogueing or targeted pesticide applications, and reduce crop losses. In the future, we plan to map the minimal epitope recognized by 22F4/45C4, express singlechain variable fragments in *Pichia pastoris* to lower production costs, and integrate the test with a smartphone reader for semi-quantitative analysis and geo-tagged disease mapping.

## Data Availability

The datasets presented in this study can be found in online repositories. The names of the repository/repositories and accession number(s) can be found in the article/supplementary material.
